# Endogenous Carbon Monoxide Signaling Modulates Mitochondrial Function and Intracellular Glucose Utilization: Impact of the Heme Oxygenase Substrate Hemin

**DOI:** 10.3390/antiox9080652

**Published:** 2020-07-23

**Authors:** David Stucki, Julia Steinhausen, Philipp Westhoff, Heide Krahl, Dominik Brilhaus, Annika Massenberg, Andreas P. M. Weber, Andreas S. Reichert, Peter Brenneisen, Wilhelm Stahl

**Affiliations:** 1 Institute of Biochemistry and Molecular Biology I, Medical Faculty, Heinrich Heine University Düsseldorf, D-40001 Düsseldorf, Germany; david.stucki@hhu.de (D.S.); julia.steinhausen@hhu.de (J.S.); heide.krahl@hhu.de (H.K.); anmas104@hhu.de (A.M.); reichert@hhu.de (A.S.R.); peter.brenneisen@hhu.de (P.B.); 2 Plant Metabolism and Metabolomics Laboratory, Cluster of Excellence on Plant Sciences (CEPLAS), Heinrich Heine University Düsseldorf, D-40001 Düsseldorf, Germany; philipp.westhoff@hhu.de (P.W.); dominik.brilhaus@hhu.de (D.B.); 3 Institute of Plant Biochemistry, Cluster of Excellence on Plant Sciences (CEPLAS), Heinrich Heine University Düsseldorf, D-40001 Düsseldorf, Germany; andreas.weber@uni-duesseldorf.de

**Keywords:** heme, heme oxygenase, substrate, respiratory chain, metabolism

## Abstract

Stress-inducible heme oxygenase-1 (HO-1) catalyzes the oxidative cleavage of heme yielding biliverdin, ferrous iron, and carbon monoxide (CO). Heme oxygenase activity has been attributed to antioxidant defense via the redox cycling system of biliverdin and bilirubin. There is increasing evidence that CO is a gaseous signaling molecule and plays a role in the regulation of energy metabolism. Inhibitory effects of CO on the respiratory chain are well established, but the implication of such a process on the cellular stress response is not well understood. By means of extracellular flux analyses and isotopic tracing, we studied the effects of CO, either released from the CO donor CORM-401 or endogenously produced by heme oxygenases, on the respiratory chain and glucose metabolism. CORM-401 was thereby used as a tool to mimic endogenous CO production by heme oxygenases. In the long term (>60 min), CORM-401-derived CO exposure inhibited mitochondrial respiration, which was compensated by increased glycolysis accompanied by a loss of the ATP production rate and an increase in proton leakage. This effect pattern was likewise observed after endogenous CO production by heme oxygenases. However, in the present setting, these effects were only observed when sufficient substrate for heme oxygenases (hemin) was provided. Modulation of the HO-1 protein level was less important. The long-term influence of CO on glucose metabolism via glycolysis was preceded by a short-term response (<30 min) of the cells to CO. Stable isotope-labeling experiments and metabolic flux analysis revealed a short-term shift of glucose consumption from glycolysis to the pentose phosphate pathway (PPP) along with an increase in reactive oxygen species (ROS) generation. Overall, we suggest that signaling by endogenous CO stimulates the rapid formation of reduction equivalents (NADPH) via the PPP, and plays an additional role in antioxidant defense, e.g., via feed-forward stimulation of the bilirubin/biliverdin redox cycling system.

## 1. Introduction

Heme oxygenases (HOs) catalyze the oxidative cleavage of heme to yield biliverdin, ferrous iron, and carbon monoxide (CO). Biliverdin is then further degraded to bilirubin via biliverdin reductase [1]. In mammalian cells, two major isoforms of heme oxygenases, HO-1 and HO-2, are present and located mainly at the endoplasmatic reticulum [2]. While HO-2 is constitutively expressed, the expression of HO-1 is triggered by the nuclear factor (erythroid derived-2)-like 2 (Nrf2)/kelch-like ECH-associated protein 1 (Keap1) system and responds to numerous exogenous and endogenous stressors, including electrophilic compounds or prooxidative stimuli. Among them are several reactive oxygen species (ROS), such as superoxide, hydrogenperoxide, and lipidperoxides, but also transition metals, ultraviolet light, secondary plant constituents, and the natural substrate of HOs, namely heme molecules [3,4,5]. In this context, an induction of HO-1 expression has been associated with the cellular stress response, in particular as an adaptive mechanism of antioxidant defense [6]. Scavenging of ROS has been attributed to the redoxcycling system of biliverdin and bilirubin, which comprises oxidation of bilirubin by ROS to yield biliverdin and enzymatically catalyzed reduction of biliverdin to bilirubin with nicotinamide adenine dinucleotide phosphate (NADPH) as an electron donor [7].

CO is a highly toxic gas, which binds after inhalation strongly to the heme moiety of hemoglobin and thus impairs oxygen transport and delivery throughout the organism [8]. However, a new aspect of research on CO covers the concept that CO acts as an endogenously produced signaling molecule in line with other gaseous compounds, such as nitric oxide or hydrogen sulfide [9]. Over the past decades, evidence has accumulated that beyond the direct antioxidant system of biliverdin/bilirubin, CO signaling contributes significantly to the HO-mediated cytoprotective effects and coordinates cellular defense. In this context, CO signaling is thought to exert protective effects for organisms and cells under conditions of sepsis, myocardial injury, and fibrosis or inflammation [10,11,12,13]. Furthermore, beneficial CO effects have been demonstrated in experimental models of multiple sclerosis [14,15,16], type I diabetes [17], and systemic lupus erythematosus [18]. Although the mechanisms of action are far from understood, protective properties of low CO doses are widely accepted. Obviously, control of endogenous CO synthesis depends on the enzyme activity of HOs and/or provision of substrate (heme), but these relations are poorly understood.

Intracellular target structures of CO are likely ferrous iron-containing proteins, in particular heme proteins, such as constituents of the respiratory chain or cytochrome P450-dependent monooxygenases [19,20]. Inhibitory effects of CO on the respiratory chain are well established; however, the implication of such a process on the stress response and on altering the energy metabolism of a cell is not well understood. The interplay of CO with the major ATP generating system and resulting shifts in the use and metabolism of energy carriers like glucose and thus the energy status of the cell likely represents a key regulatory hub.

For studying the effects of low CO amounts on cellular processes, CO-releasing molecules (CORMs) have been introduced as valuable research tools [21,22]. CORMs provide a continuous exposure of cells to standardized amounts of CO (in contrast to the application of gaseous CO). From the number of different CORMs, CORM-401 was already shown to be a suitable CO delivery system, especially for studies on the impact of CO on energy metabolism [23]. CORM-401 releases CO only in the close presence of suitable CO acceptors (heme proteins) [24] and therefore ensures controlled intracellular availability of the signaling molecule. In this study, we used CORM-401 as a CO delivery system to mimic effects on cellular energy metabolism and related processes by endogenously generated CO.

## 2. Materials and Methods 

### 2.1. Chemicals

Tetracarbonyl[N-(dithiocarboxy-κS,κS’)-N-methylglycine]manganite(CORM-401), Dulbecco’s Modified Eagle’s Medium (DMEM, low glucose), manganese sulfate monohydrate, hemin, DMSO, hemin, [U-^13^C]-glucose, and PBS were from Sigma-Aldrich (Deisenhofen, Germany); methanol and acetonitrile from Merck (Darmstadt, Germany); trifluoroacetic acid and chloroform were from VWR (Langenfeld, Germany); sodium dodecyl sulfate (SDS) and Tris-(hydroxymethyl)-aminomethan from Roth (Karlsruhe, Germany); and sarcosine-N-dithiocarbamate was obtained from 3B Scientific Corporation (Wuhan, China). All compounds used in the mito stress test or glycolytic rate assay were from Agilent Technologies (Santa Clara, CA, USA).

### 2.2. Handling of CORM-401 and iCORM-401 

CORM-401 was freshly dissolved in PBS (5 mM) and aliquots were stored at −20 °C. The compound is stable under these conditions [23]. CO release from CORM-401 occurs only in the presence of CO acceptors, such as heme proteins [24,25]. In accordance with the literature, an equimolar mixture of sarcosine-N-dithiocarbamate and MnSO_4_ was used as iCORM-401, the inactive form of CORM-401, as a control for non-CO related effects of CORM-401 [24,26,27]. CORM-401 and iCORM-401 were protected from light at all times.

### 2.3. Cell Culture

Murine embryonic fibroblasts (MEFs) (a kind gift from Dr. Ishihara [28]), human hepatocellular carcinoma (HepG2) cells (85011430, Sigma-Aldrich, Deisenhofen, Germany), normal human dermal fibroblasts (NHDFs) (C-12300, Promocell, Heidelberg, Germany), and human mammary gland breast cancer (MCF-7) cells (HTB-22, ATCC, Wesel, Germany) were cultured in DMEM (low glucose), supplemented with 10% fetal bovine serum (FBS, PAN-Biotech, Aidenbach, Germany), penicillin (100 U/mL), streptomycin (100 µg/mL), and GlutaMAX™ (2 mM) at 37 °C and 5% CO_2_ in a humidified atmosphere.

All cells were washed and supplied with XF assay medium (see next paragraph) for 1 h prior to treatment. Cell confluency was about 70–90% before the start of experiments. If not stated otherwise, treatment was also performed using XF assay medium. 

### 2.4. Extracellular Flux Analyses

Extracellular flux (XF) analyses were performed with an XFe96 analyzer (Agilent Technologies, Santa Clara, CA, USA). All XF analyses were performed in a DMEM-based XF assay medium (103575-100, Agilent Technologies, Santa Clara, CA, USA) supplemented with glucose (1 g/L), GlutaMAX™ (2 mM) and sodium pyruvate (1 mM), but without FBS, antibiotics, and phenol red. For experiments 7500 MEFs, 12,000 NHDFs, 15,000 HepG2 or 50,000 MCF-7 cells per well were plated in XFe96 well plates and incubated for 24 h in normal cell culture medium at 37 °C and 5% CO_2_. After culture medium was changed, cells were placed in a non-CO_2_ incubator (37 °C) for 1 h prior to experiments. A XFe96 sensor cartridge was loaded with 20 µL of CORM-401 or iCORM-401 solutions or medium (control). Final concentrations of compounds in the wells were 50 µM. During the experiment, the oxygen consumption rate (OCR) and extracellular acidification rate (ECAR) were determined in consecutive measurement cycles. One measurement cycle consisted of 3 min mixing and 3 min measuring.

Next to the basic XF analyses described above, the so-called “mito stress test” was performed for further characterization of the effects on cellular respiration. In the mito stress test, modulators of the respiratory chain are injected in the order: oligomycin (MEF and NHDF: 1 µM; HepG2 and MCF-7: 2 µM), carbonyl cyanide-*p*-trifluoromethoxyphenylhydrazone (FCCP) (MEF, NHDF and HepG2: 1 µM; MCF-7: 0.5 µM), rotenone + antimycin A (0.5 µM each). Differences in OCR levels between injections give the parameters maximal respiration, ATP production, proton leak (here the term “proton leak” is used to describe the process of protons moving from the mitochondrial intermembrane space into the matrix independently of ATP synthase), and non-mitochondrial respiration. Maximal respiration is defined as the highest OCR value after FCCP injection minus non-mitochondrial respiration (OCR after rotenone + antimycin A injection). ATP production is defined as the difference of the last OCR value before oligomycin injection and the lowest OCR value after oligomycin injection but before FCCP injection. Proton leak is defined as the lowest OCR value after oligomycin but before FCCP injection minus non-mitochondrial respiration. The mito stress test was either performed after acute injection of CORM-401/iCORM-401 or acute KCN injection or after 4 h of pre-incubation with the heme oxygenase substrate hemin. Upon dilution in aqueous solutions, hemin decomposes to Cl^−^ and heme, the natural substrate of heme oxygenases. Cells were then stained for DNA amount with SYBR™ green according to the manufacturer’s instructions (ThermoFisher) and fluorescence signal (λ_ex_: 492 nm/λ_em_: 520 nm) was measured with a plate reader (Tecan, Infinite M200). Data achieved after pre-incubation of cells with hemin was normalized to SYBR™ green fluorescence. In another set of experiments using MEFs, first oligomycin was added to reaction wells and then 50 µM CORM-401/iCORM-401 or medium for control. OCR and ECAR levels were then monitored over time.

Another assay performed with XF technology is the glycolytic rate assay. The glycolytic rate assay provides information about basal glycolytic rates as well as glycolytic compensatory capacities upon inhibition of the mitochondrial respiratory chain. Extracellular acidification after acute injection of CORM-401/iCORM-401 or medium only (control) gives the induced glycolysis. Following this, rotenone + antimycin A (0.5 µM each) were injected, provoking a glycolytic compensation for inhibition of mitochondrial respiration (compensatory glycolysis). Addition of 2-deoxyglucose (55 mM) inhibits hexokinase and was used to verify that extracellular acidification is glycolysis-based.

### 2.5. Transfection of Cells and Gene Silencing

MEFs were transfected 24 h prior to experiments via reverse transfection. For overexpression experiments, 1 µg pCMV3-Hmox1 or the corresponding empty vector (MG52753-UT and CV011, Sino Biological, Eschborn, Germany) and Lipofectamine™ 2000 transfection reagent (ThermoFisher, Schwerte, Germany) were mixed in a reaction tube for 10 min to form transfection complexes. Transfection complexes were then gently mixed with a cell suspension of 100,000 MEFs per mL and cells were subsequently plated on appropriate dishes and plates.

For gene silencing experiments, RNAiMAX transfection reagent (ThermoFisher) was mixed with Hmox1 siRNA mix (GS15368) or negative siRNA (SI03650318, Qiagen, Hilden, Germany) in a reaction tube (final concentration of siRNA: 20 nM), and incubated for 10 min to allow formation of reaction complexes. Transfection complexes were then gently mixed with an MEF cell suspension (100,000 cells/mL) and cells were plated on appropriate dishes and plates.

### 2.6. SDS PAGE and Western Blot Analysis

For Western blot analysis, cells were lysed with 1% SDS, supplemented with a protease inhibitor cocktail (Roche, Grenzach, Germany). Protein determination was performed using the DC™ protein assay kit from Biorad (Munich, Germany). Equal amounts of total protein (∼30 μg) were subjected to SDS-PAGE (12% gels), followed by electro blotting of proteins onto polyvinylidene difluoride (PVDF) membranes (pore size 0.45 µm, GE Healthcare, Solingen, Germany). Blots were developed with the ECL system provided by Cell Signaling Technology (Frankfurt a. Main, Germany) and chemiluminescence signals were monitored using the Fusion SL Advance gel documentation device (Peqlab, Erlangen, Germany). Primary antibodies from rabbits were used: Anti-heme oxygenase-1 (ab52947) was from Abcam (Cambridge, UK); anti-β-tubulin (9F3), anti-hexokinase I (C64G5), anti-GAPDH (D16H11), and anti-pyruvatedehydrogenase (PDH; C54G1) from Cell Signaling (Leiden, Netherlands). Secondary antibodies were horseradish peroxidase (HRP)-coupled goat anti-rabbit IgG (111-035-144) from Dianova (Hamburg, Germany). Equal loading of gels was confirmed using signals from β-tubulin or coomassie blue staining.

### 2.7. Metabolomics 

About 1.8 × 10^6^ MEFs per dish were plated on 10-cm dishes 48 h prior to the experiment. Cells were then supplied with XF assay medium for 1 h containing 1 g/L [U-^12^C]-glucose. Treatment of cells with 50 µM CORM-401/iCORM-401 or medium only (control) was then performed in XF assay medium containing 1 g/L [U-^13^C]-glucose. Before the start of the incubation (0 min) and at time points of 30, 90 and 240 min, cells were washed with ice cold PBS, resuspended in PBS, and transferred to reaction tubes. Samples were then centrifuged at 800 *g* and 4 °C for 5 min. The supernatant was discarded, and the cell pellet resuspended in 500 µL 22% water, 22% chloroform, and 46% methanol (*v*/*v*/*v*) for lysis of cells. Samples were then centrifuged at 14,000 *g* at 4 °C for 10 min and 100 µL of the supernatant (extract) were transferred to a glass inlet and dried by lyophilization for ion chromatography—mass spectrometry (IC-MS) analysis. The residual 400 µL were stored at −80 °C as a sample backup. For IC-MS measurements, a combination of a Dionex ICS-6000 HPIC and a Thermo Fisher Scientific Q Exactive Plus mass spectrometer was used according to a method described by others previously [29] with slight modifications. In brief, the dried sample was reconstituted in 100 µL of deionized water of which 5 µL were injected via a Dionex AS-AP autosampler. For the anion exchange chromatography, a Dionex IonPac AS11-HC column (2 mm × 250 mm, 4 μm particle size, Thermo Fisher Scientific) equipped with a Dionex IonPac AG11-HC guard column (2 mm × 50 mm, 4 μm, Thermo Fisher Scientific) was used and the mobile phase was generated using an eluent generator with a potassium hydroxide cartridge starting at 10 mM KOH. The mass spectrometer operated in negative mode with a combination of full mass scan and a data-dependent Top5 MS2 (ddMS2) experiment with a resolution of 140,000 and 17,500 respectively. Data analysis was conducted using Compound Discoverer (version 3.1, Thermo Fisher Scientific). The standard workflow for stable isotope labeling from Compound Discoverer was chosen and the default settings were used, which are 5 ppm mass tolerance, 30% intensity tolerance, and 0.1% intensity threshold for isotope pattern matching. As an additional level of validation, an in-house database for retention times and MS^2^ spectra was created using mzVault (Thermo Fisher Scientific) and implemented in the annotation workflow. 

### 2.8. Analysis of 2-Hydroxyethidium Formation via HPLC

About 2 × 10^5^ MEFs per dish were plated on 6 cm dishes 24 h prior to the experiment. Cells were then washed with Hank’s Balanced Salt Solution (HBSS) and loaded with 20 µM dihydroethidium (DHE) in HBSS for 30 min. Cells were washed again and treated with 50 µM CORM-401 or iCORM-401 in HBSS or HBSS only (control) for 30 min. Subsequently, cells were washed with ice-cold PBS and resuspended in 1 mL of PBS. Samples were centrifuged at 800 *g* for 3 min at 4 °C. The supernatant was discarded and the pellet resuspended in 200 µL of methanol/acetonitrile/water/TFA (40/40/20/0.1; *v*/*v*/*v*/*v*) for lysis of cells, and centrifuged at 14,000 *g* for 10 min at 4 °C. Cell pellets were kept for protein determination as described previously with slight modifications [30]. Briefly, cell pellets were air dried, resuspended in 63 mM Tris/HCl buffer (pH 6.8) containing 2% SDS, and stored at −20 °C until further use. Samples were then sonicated, and the total protein amount was determined as described above. The HPLC system consisted of a Merck-Hitachi L-7100 pump connected with a fluorescence detector (Merck-Hitachi L-7480) and a data registration system. HPLC was performed with a mobile phase of 21% acetonitrile, 79% water, and 0.1% TFA (*v*/*v*/*v*) at a flow rate of 2.0 mL/min. A Suplex™ pKb-100 column (250 × 4.6 mm, 5 µm) from Sigma-Aldrich was used as the stationary phase. Cellular extracts were diluted 1:2 with the mobile phase and directly injected into the HPLC system. 2-Hydroxyethidium (2-OH-E^+^) was measured at λ_ex_ = 480 nm/λ_em_ = 580 nm and a retention time of about 9 min. Signal areas were normalized to the total protein amount and the control, which was set to 1.

### 2.9. Statistical Analysis

If not stated otherwise, data is given as the mean of three independent experiments (n = 3) and error bars represent the standard deviation (SD). Data were analyzed either using unpaired Student’s t-test or one-way ANOVA, with *p* ≤ 0.05 being considered a statistically significant difference.

## 3. Results

### 3.1. Extracellular Flux Analysis Shows a Distinct Modulation of Cellular Respiration and Glycolysis by CO

**Figure 1:**
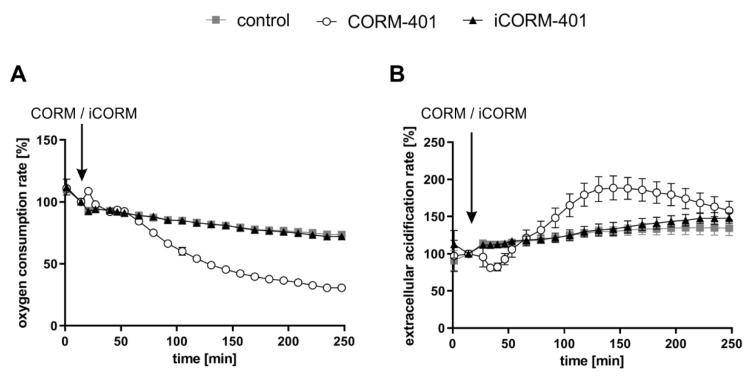
Extracellular flux technology reveals CO-dependent modulation of cellular respiration and extracellular acidification by CORM-401. CORM-401 and iCORM-401 were injected (indicated by arrows) into reaction wells and OCR (**A**) and ECAR (**B**) were measured over time. Final concentration of compounds: 50 µM. Three independent experiments were performed (n = 3). Representative OCR and ECAR curves are shown. Data represent mean ± SD of at least six technical replicates. OCR = oxygen consumption rate, ECAR = extracellular acidification rate.

CO is known to inhibit cellular respiration via binding to heme proteins of the mitochondrial electron transport chain. Thus, we first examined in MEFs the effects of CORM-401 and inactive CORM-401 (iCORM-401) on cellular respiration and extracellular acidification (as a marker for glycolysis) by means of extracellular flux technology. Cellular response to CORM-401 administration followed a time-dependent two-phase profile. In the first phase (<60 min), directly after the application of 50 µM CORM-401, a moderate and transient increase of the cellular oxygen consumption rate (OCR) (third measurement point in Figure 1A) occurred. In the second phase (>60 min), a continuous decrease of OCR was observed as expected upon inhibition of mitochondrial respiration by CO (Figure 1A), confirming the validity of the experimental setting for MEFs. Concomitantly, the extracellular acidification rate (ECAR) transiently decreased within the first 30 min after CORM-401 injection and only increased above the control at later time points (>60 min) (Figure 1B). Treatment of cells with iCORM-401 did not affect OCR and ECAR compared to the control.

### 3.2. The Mito Stress Test Reveals a CO-Specific Effect Pattern after Treatment of Different Cell Types with CORM-401

**Figure 2:**
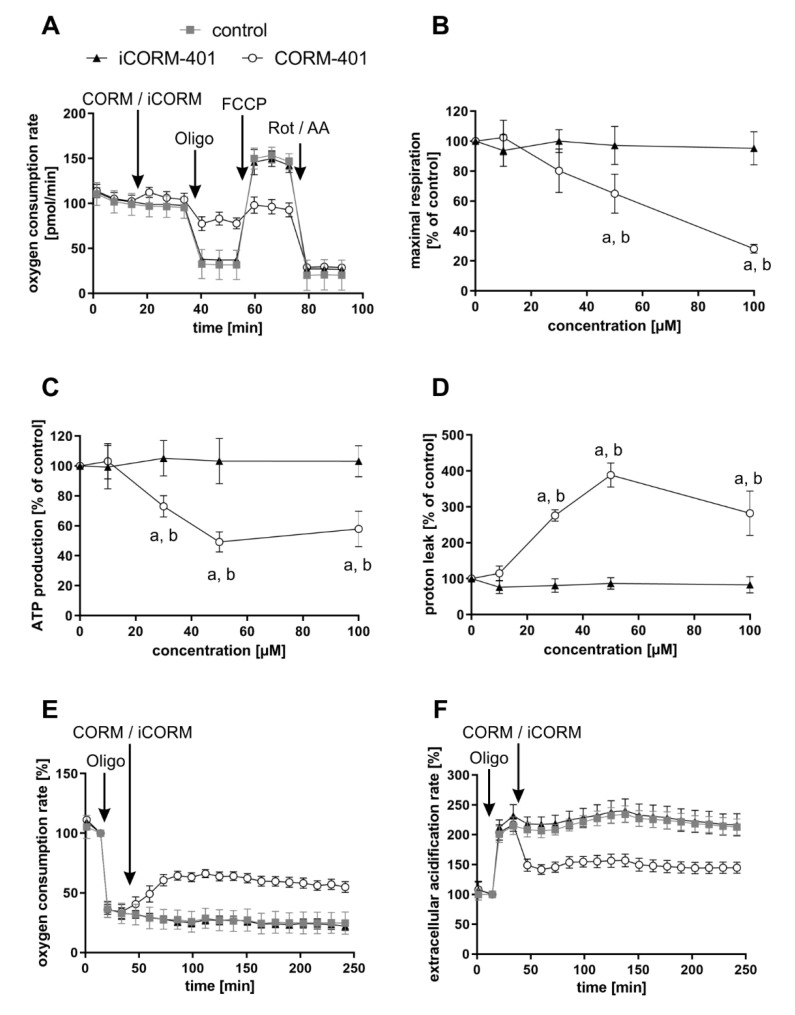
Modulation of mitochondrial respiratory parameters by CO results in a specific effect pattern. CORM-401 and iCORM-401 were injected into reaction wells and the mito stress test (sequential injection of oligomycin, FCCP, and rotenone + antimycin A) was performed. Injections are indicated by arrows. Representative OCR curves are given (**A**) with the final concentration of CORM-401/iCORM-401: 50 µM. Data represent mean ± SD of at least six technical replicates. Quantification of the mitochondrial respiratory parameters maximal respiration (**B**), ATP production (**C**), and proton leak (**D**) is shown for 10–100 µM CORM-401/iCORM-401. Data represent mean ± SD of three independent experiments (n = 3). In a modified experimental setting, first oligomycin and then 50 µM CORM-401 or iCORM-401 were applied and OCR (**E**) and ECAR (**F**) were monitored over time. Representative OCR and ECAR curves of three independent experiments are shown. Data represent mean ± SD of at least six technical replicates. OCR = oxygen consumption rate, ECAR = extracellular acidification rate, Oligo = oligomycin, Rot/AA = rotenone + antimycin A. Statistical analysis was performed using one-way ANOVA and detailed results are given in Table S1. a: control vs. CORM-401, b: CORM-401 vs. iCORM-401 with *p* ≤ 0.05.

Modulation of mitochondrial function by CORM-401 during the first phase (<60 min) after the start of exposure was further analyzed with the mito stress test. In this assay, modulators of the mitochondrial electron transport chain are sequentially added to cells and the parameters ATP production, maximal respiration, or proton leakage (for details, see the materials and methods section) can be calculated from the differences in OCR levels before and after the addition of the compounds. CORM-401, iCORM-401, or medium (control) were injected into the reaction wells directly before the mito stress test was started. Directly after the application of CORM-401 (50 µM), cellular OCR was slightly increased (Figure 2A), confirming the results from experiments shown in Figure 1A. Subsequent inhibition of the ATP synthase by oligomycin only led to a moderate drop of cellular OCR, according to a decreased mitochondrial ATP production in the CORM-401 treatment group, as compared to the control. Addition of the uncoupling agent FCCP to the test system increased OCR and the increase is a measure for maximal respiration of cells. In comparison to iCORM-401 and control, maximal respiration was lower in cells treated with CORM-401. With the final application of rotenone and antimycin A, inhibitors of complex I and III, respectively, the electron transport chain is completely inhibited. The remaining OCR reflects only oxygen-consuming processes not related to the mitochondrial respiratory chain. Here, no major differences were found between treatment groups. 

Coupling of substrate oxidation and ATP synthesis is usually not complete, as protons move from the mitochondrial intermembrane space into the matrix independently of ATP synthase. Such proton transfer processes are collectively termed “proton leak” and can be calculated in the present setting from the difference of OCR after oligomycin injection and after rotenone and antimycin A injection. Proton leak in cells treated with CORM-401 was dramatically increased whereas iCORM-401 treatment had no effect (Figure 2D). A schematic depiction of the OCR curves from Figure 2A and the resulting parameters is given in Figure S1. 

The quantification and dose dependency of the parameters described above are summarized in Figure 2B–D and statistically significant differences are given in Table S1. None of the parameters were affected by the lowest concentration of CORM-401, indicating 10 µM as a threshold concentration for CO activity of the compound. At higher levels (30–100 µM), CORM-401 significantly decreased maximal respiration in a dose-dependent manner, to a residual activity of about 30% of the control at 100 µM (Figure 2B). ATP production also concentration dependently decreased upon CORM-401 treatment. After the treatment of cells with 50 µM CORM-401, ATP production was lowered by 50%; however, no further decrease was found at the 100 µM level (Figure 2C). Opposing this, proton leak was significantly increased following CO exposure (Figure 2D). At 50 µM CORM-401, proton leak was about 4-fold higher compared to the control, but this effect was not enhanced at 100 µM. A CO-dependent induction of proton leakage by CORM-401 was also demonstrated in another experimental setting. First, ATP synthase was inhibited by oligomycin injection, leading to a direct decrease of cellular OCR (Figure 2E) and a compensatory increase of ECAR (glycolysis) (Figure 2F). Next, CORM-401, iCORM-401, or medium only (control) were added to the reaction wells. While no further change of OCR and ECAR was observed under control and iCORM-401 conditions, CORM-401 led to an increase of OCR levels. Since this increase of respiration is independent of ATP synthase activity, an induction of proton leakage by CO is suggested. Simultaneously, CORM-401 exposure lowered ECAR levels, which were elevated as a compensatory mechanism for the oligomycin-mediated inhibition of ATP synthase.

To demonstrate the general validity of the observed CO effects on parameters of mitochondrial respiration, three more cell types (NHDFs, HepG2 and MCF-7 cells) were subjected to the mito stress test with CORM-401 under the same conditions and almost similar results were observed (Figure S2). For further control, MEFs were subjected to the mito stress test after acute KCN injection. Comparable to CO, cyanide is known to inhibit mitochondrial respiration via binding to the heme moiety of cytochrome *c* oxidase [31]. Increasing concentrations of cyanide decreased maximal respiration and mitochondrial ATP production. However, no effect on proton leakage was found (Figure S3), even if the respiratory chain was not completely inhibited.

In summary, our results demonstrate that modulation of mitochondrial function by CO occurs very fast and presents both inhibitory (decrease of maximal respiration and ATP production) and stimulating (increase of proton leak) effects with respect to cellular respiration. In the first phase after CORM-401 exposure (<60 min), inhibitory and stimulating effects likely counteract each other and no major effects on basal OCR are found, whereas in the second phase (>60 min), inhibitory effects are predominating and total cellular OCR is lowered.

### 3.3. Substrate Availability is Involved in the Regulation of Endogenous CO Production

**Figure 3:**
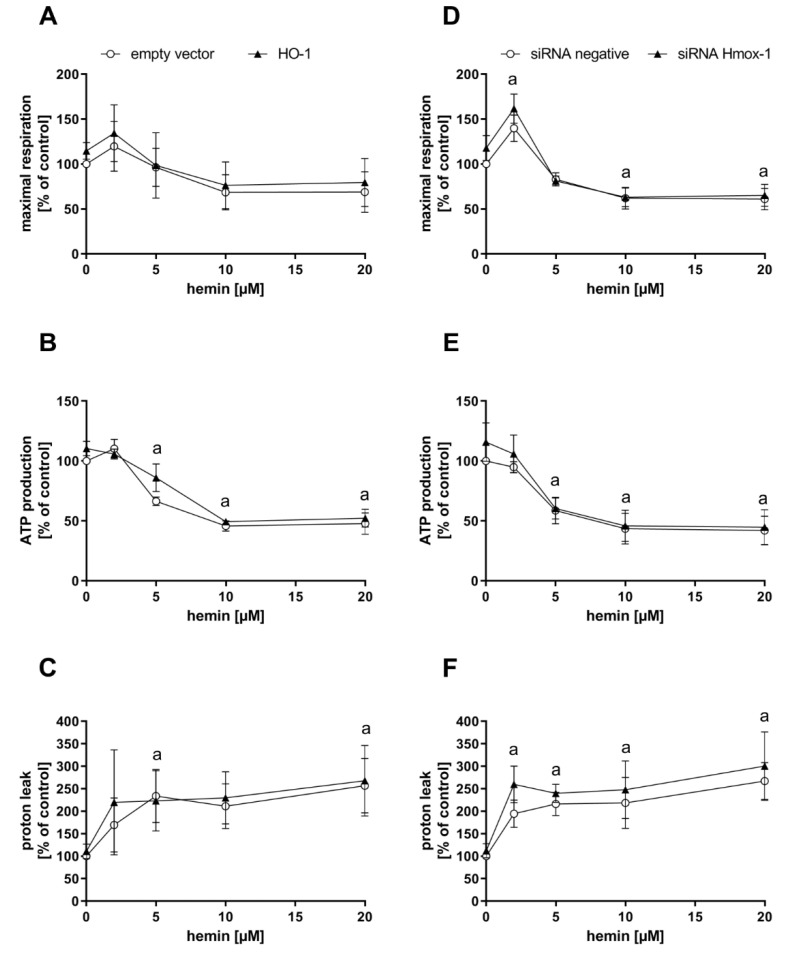
Hemin treatment of cells leads to an effect pattern of mitochondrial respiratory parameters comparable to CO. Heme oxygenase-1 protein levels of MEFs were genetically modified by either overexpression (left panel) or knockdown using siRNA (right panel). Cells were then treated with 2–20 µM hemin for 4 h and subjected to the mito stress test. Quantifications of the mitochondrial respiratory parameters maximal respiration (**A**,**D**), ATP production (**B**,**E**), and proton leak (**C**,**F**) are given. Data represent the mean ± SD of three independent experiments (n = 3). Statistical analysis was performed using one-way ANOVA and detailed results are given in Table S2. a: control vs. treatment with *p* ≤ 0.05.

Endogenously, CO is produced upon degradation of heme by heme oxygenases, with HO-1 as a stress-inducible form. To test whether CO-dependent effects observed after CORM-401 treatment can be recapitulated with endogenously generated CO, MEFs were transfected with a plasmid encoding for the *Hmox1* gene (HO-1 group) or an empty vector for the control. HO-1 protein levels were elevated in the HO-1 group compared to the control as confirmed by Western blot analysis (Figure S4A). Transfected cells were then preincubated with different concentrations of hemin (substrate of heme oxygenases) or medium with 0.1% DMSO (control) for a period of 4 h and then subjected to the mito stress test (Figure 3A–C). In accordance with the previous results obtained with the CO donor CORM-401, maximal respiration and ATP production were decreased, and proton leak increased in a concentration-dependent manner between 5 and 20 µM of hemin. At low concentrations of hemin (2 µM), maximal respiration slightly increased in both groups (Figure 3A). While no major effect on ATP production of cells was found at 2 µM hemin, treatment with higher concentrations (10 and 20 µM) led to a significant decrease of about 50% compared to the untreated control (Figure 3B). Proton leak was found to be elevated at 2 µM hemin to about 160–200% of the control, but this effect was hardly increased at higher concentrations of hemin (Figure 3C). The effects in response to hemin treatment were similar in both groups (empty vector and HO-1).

In the following experiment, cells were transfected with siRNA against *Hmox1* encoded mRNAs or negative siRNA. Depletion of the HO-1 protein amount was confirmed by Western blot analysis, with β-tubulin as the loading control (Figure S4B). Cellular maximal respiration was significantly increased with 2 µM hemin (140–160% of control), but concentration-dependently decreased below the control level at 5–20 µM hemin (Figure 3D). No effect on ATP production was found after incubation of cells with 2 µM hemin, while a decrease to about 60% of the control level was observed at the 5 µM level, which was further decreased to about 45% at concentrations above 10 µM hemin (Figure 3E). The proton leak of cells was significantly increased after treatment with 2 µM hemin to about 200–250% and moderately further increased to about 270% at the 20 µM level (Figure 3F). No major difference was found between both groups (negative siRNA and *Hmox1* siRNA) upon hemin treatment.

In summary, the CO-derived effect pattern in the mito stress test after exposure of cells to the model substance CORM-401 was also observed when cells were supplied with the HO substrate hemin, suggesting endogenous CO production. While HO-1 enzyme levels were not limiting under these conditions, sufficient provision of the substrate was demanded to obtain CO effects.

We thereby demonstrated the suitability of CORM-401 to mimic endogenous CO production by heme oxygenases and hence decided to use CORM-401 in the following experiments as a model compound for further analysis of the CO effects on cellular metabolism.

### 3.4. CO Lowers Compensatory Glycolysis of Cells

Upon inhibition of the mitochondrial respiratory chain, less ATP is produced, which is usually accompanied by a compensatory stimulation of glycolysis. In accordance with the inhibitory effect on mitochondrial respiration (Figure 1A, >60 min), we determined a decreased mitochondrial ATP production after CORM-401 treatment (Figure 2 and Figure S2). In the long term (>60 min), we also observed a compensatory increase in ECAR a marker for glycolytic flux (Figure 1B). However, in the first phase (about 30 min) after CORM-401 addition, ECAR was transiently decreased, an observation made in numerous experiments with different cells.

**Figure 4:**
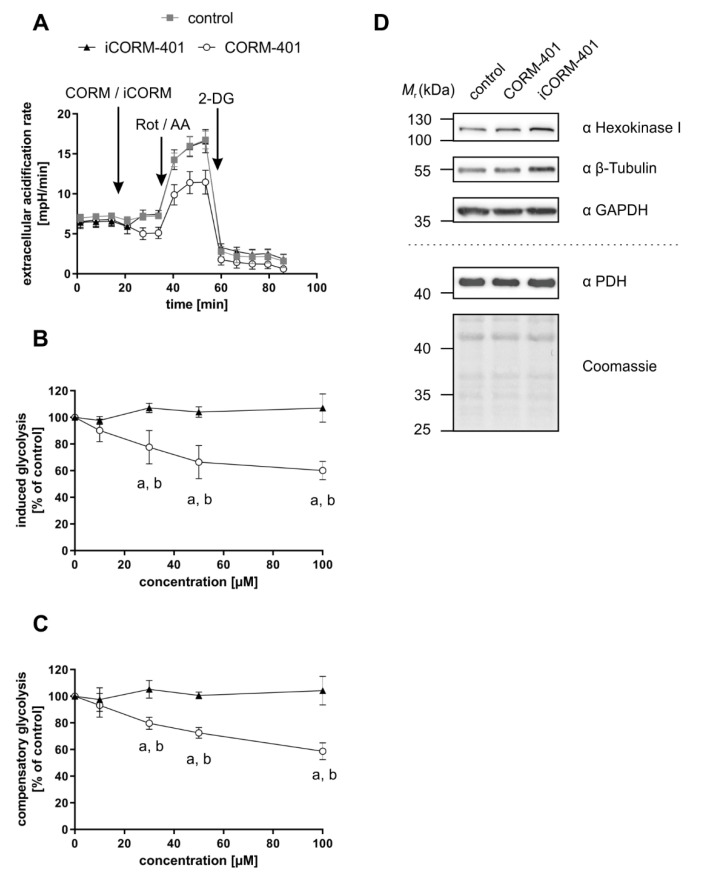
CO-dependent decrease of glycolytic parameters is independent of glycolysis enzyme protein levels. CORM-401 and iCORM-401 were injected into reaction wells and the glycolytic rate assay (sequential injection of rotenone + antimycin A and 2-DG) was performed. Injections are indicated by arrows. Representative ECAR curves are given (**A**) with a final concentration of CORM-401/iCORM-401: 50 µM. Data represent mean ± SD of at least six technical replicates. Quantifications of the glycolytic parameters compensatory glycolysis (**B**) and induced glycolysis (**C**) are shown for 10–100 µM CORM-401/iCORM-401. Data represent mean ± SD of three independent experiments (n = 3). Representative Western blot analyses of glycolysis enzymes after treatment of MEFs with 50 µM CORM-401/iCORM-401 or medium only for 30 min are shown (**D**). The dashed line separates results of two different gels, loaded with the same samples (n = 3). ECAR = extracellular acidification rate, Rot/AA = rotenone + antimycin A, 2-DG = 2-deoxyglucose, PDH = pyruvate dehydrogenase. Statistical analysis was performed using one-way ANOVA and detailed results are given in Table S3. a: control vs. CORM-401, b: CORM-401 vs iCORM-401 with *p* ≤ 0.05.

To further study this early effect, a glycolytic rate assay was performed applying CORM-401, iCORM-401, or medium (control) directly before the start of the test. Upon application of CORM-401 (50 µM), ECAR directly decreased below the control level (Figure 4A), which confirms the results of an acute response (<30 min) mentioned above. Such an acute response is termed “induced glycolysis” and this parameter was significantly lowered by CORM-401 treatment in a concentration-dependent manner. While 10 µM CORM-401 had no effect, about 60% residual activity compared to the control was found at the 100 µM level (Figure 4B). Next, rotenone and antimycin A were injected to abolish electron transfer in the mitochondrial respiratory chain. This treatment induces a compensatory increase of glycolysis accompanied by elevated ECAR levels. However, this compensatory effect was less pronounced in CORM-401-treated cells (Figure 4A) and dose-dependently decreased at concentrations of CORM-401 above 10 µM (Figure 4C). Since the application of iCORM-401 did not affect parameters of the glycolytic rate assay compared to the control, this modulation of glucose metabolism is triggered by CO, which was also reflected in the statistical analysis of the data (Table S3), showing significant differences between the CORM-401 and iCORM-401 treatment groups.

Changes in the protein levels of glycolytic enzymes do not play a role in this context. Following incubation of cells with 50 µM CORM-401 or iCORM-401, no change in the protein levels of hexokinase I, GAPDH, or PDH were observed by Western blot analysis (Figure 4D). Equal loading of the gels was controlled via β-tubulin analysis or coomassie blue staining, respectively. 

Thus, this short-term decrease in glycolysis implies other limiting factors.

### 3.5. CO Causes a Transient Metabolic Shift from Glycolysis to Pentose Phosphate Pathway

**Figure 5:**
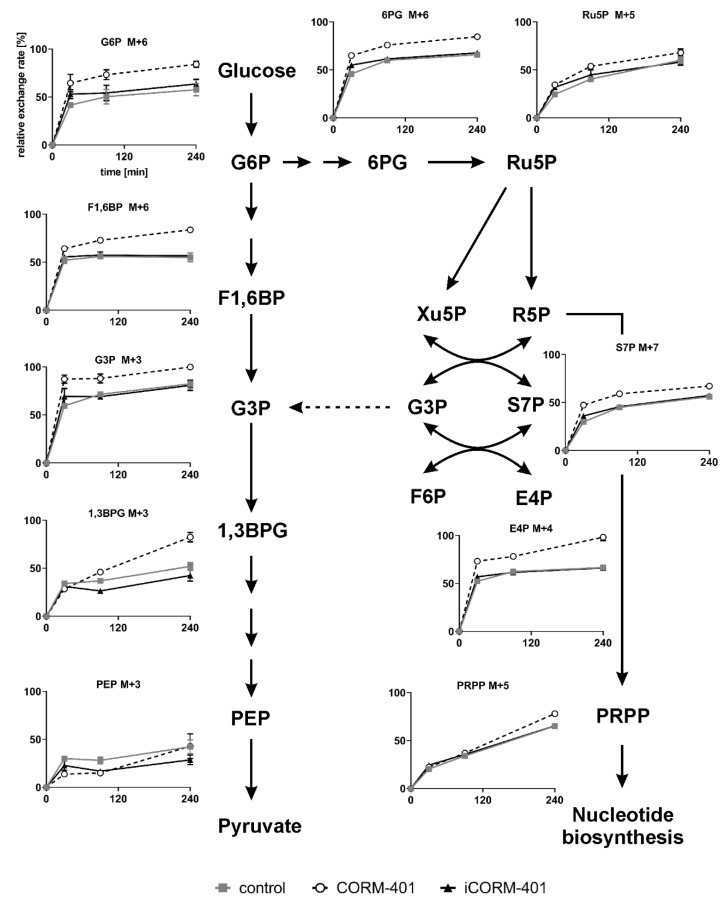
Scheme of the glycolysis and pentose phosphate pathway and the uniform metabolite labeling after simultaneous incubation of cells with [U-^13^C]-glucose and CORM-401/iCORM-401. MEFs were simultaneously treated with [U-^13^C]-glucose and 50 µM CORM-401/iCORM-401 or medium only (control), for 30, 90 or 240 min. Cells were then lysed and extracts analyzed via IC-MS analysis. Relative exchange rates of uniformly labeled metabolites are shown as mean ± SD (n = 4). Statistical analysis was performed using one-way ANOVA and results are given in Table S4.

Since the CO-mediated fluctuations of ECAR could not be explained by changes of the protein level, glycolytic activity was assessed by isotopic tracing with [U-^13^C]-glucose. Cells were loaded with labeled glucose and then treated with 50 µM CORM-401, iCORM-401, or medium only (control). The metabolic pattern was analyzed by means of IC-MS, 30, 90 and 240 min after the addition of CORM-401. Relative exchange rates of uniformly labeled metabolites are shown in Figure 5 and statistical analysis of the data using one-way ANOVA is given in Table S4.

The extracellular acidification rate (ECAR) as a marker for glycolytic flux is a read out related to the final steps of glycolysis. Among the metabolites analyzed by isotopic tracing, phosphoenolpyruvate (PEP) is most downstream in glycolysis. After 30 min of CORM-401 treatment, cells exhibited significantly less labeled PEP (M + 3), compared to control, showing a decreased flux through glycolysis within the first 30 min of CO exposure. Although at later time points (240 min), PEP (M + 3) levels are the same for control and CORM-401 treated cells, the flux is nevertheless increased, indicated by a steeper slope of the CORM-401 curve (dashed line), substantiating the observation from Figure 1B. 

Upstream of PEP, this effect is less pronounced. The relative exchange rate of 1,3-bisphosphoglycerate (1,3BPG) M + 3 at 30 min is similar for the treatment and control but is significantly higher upon CORM-401 treatment at later time points (90 or 240 min). Again, the CORM-401 curve exhibits a steeper slope, indicating an increased flux at the late time points.

The ^13^C label pattern of metabolites in the preparatory phase of glycolysis, such as fructose-1,6-bisphosphate (F1,6BP) or glucose-6-phosphate (G6P), differs from that observed in the pay-off phase. For both metabolites, higher exchange rates are measured already within 30 min of incubation with CORM-401 compared to the control. At later time points, this effect is persistent but not further enhanced as all curves (treatments and control) run in parallel between 30 and 240 min. 

Taken together, these findings suggest that glycolytic flux within the first 30 min of incubation with CORM-401 is lower in the pay-off phase and higher in the preparatory phase. Beyond 30 min, the glycolytic flux is elevated upon CO exposure.

It is conspicuous that the labeling of glyceraldehyde-3-phosphate (G3P) does not fit into this pattern. Already at an early time point (30 min) of treatment with CORM-401, a relative exchange rate of about 90% was determined for G3P M + 3 whereas the exchange rate was only 60% for F1,6BP M+6. Since G3P can also be generated in the pentose phosphate pathway (PPP), it is suggested that both pathways (glycolysis and PPP) contribute to the formation of labeled G3P M + 3. The assumption is fostered by the analysis of other metabolites of the PPP. 

Typical metabolites, such as 6-phosphogluconate (6PG), ribulose-5-phosphate (Ru5P), sedoheptulose-7-phosphate (S7P), or erythrose-4-phosphate (E4P), exhibit an increased labeling after CORM-401 exposure (30 min), compared to the control. Again, this effect is persistent but not further enhanced as the curves (treatment and control) extend parallel between 30 and 240 min. 

In summary, the data provide evidence that upon application of CORM-401, CO immediately causes a transient increase of glucose flux through the PPP, redirecting labeled fragments towards glycolysis via G3P.

It is noteworthy that the relative exchange rates for M + 5 of phosphoribosyl pyrophosphate (PRPP) do not differ between the treatment and control at time points up to 90 min. This suggests that the quick physiological response of the PPP triggered by CO mainly aims to enhance the regeneration or production of NADPH. This is often observed as a compensatory mechanism in defense of oxidative stress, providing NADPH as a cofactor for glutathione reductase for the maintenance of reduced glutathione.

### 3.6. Exposure of Cells to CORM-401/CO is Associated with a Moderate Increase of Reactive Oxygen Species

**Figure 6:**
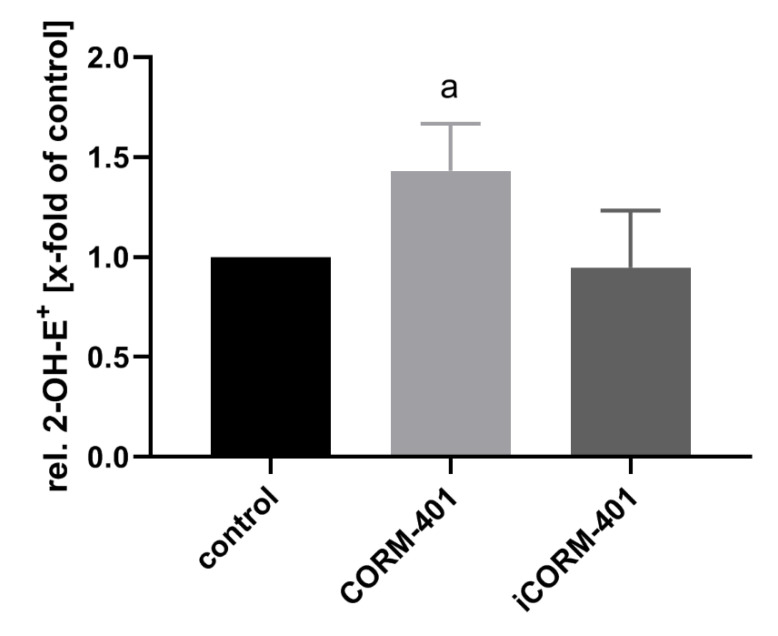
CO from CORM-401 produces a mild oxidative stress. Cells were pre-incubated with 20 µM DHE for 30 min and then treated with 50 µM CORM-401/iCORM-401 for 30 min. Formation of 2-OH-E+ as a marker for superoxide production was monitored using HPLC analysis, quantified via the signal area, and normalized to the total protein amount of cells. Data is given as the relative amount of 2-OH-E+ (control was set to 1) and represents the mean ± SD of three independent experiments (n = 3). Statistically significant differences were evaluated using Student’s t-test with a: control vs CORM-401 and *p* ≤ 0.05. DHE = dihydroethidium.

Electron leakage of the respiratory chain is a major endogenous source of reactive oxygen species, such as superoxide and hydrogen peroxide. It has been described that the inhibition of mitochondrial respiration by CO is associated with an increased production of ROS [19]. ROS in turn are known inducers of the PPP [32] in order to address the NADPH demand for antioxidant defense systems. Therefore, we investigated whether in the present setting, CORM-401 application leads to an increase in ROS levels. As a measure of ROS formation, oxidation of dihydroethidium (DHE) to 2-hydroxyethidium (2-OH-E^+^) was determined by means of HPLC. As shown in Figure 6, about 50% more 2-OH-E^+^ was found, in cells treated for 30 min with 50 µM CORM-401, compared to the control and iCORM-401 treatment group.

## 4. Discussion

Induction of HO-1 is mediated by numerous prooxidative stressors from different sources. Among them are physical noxae (radiation), secondary plant constituents, drugs, or pollutants, but also endogenous oxidative challenges related to cell or tissue responses following, e.g., lesions or infections [10,12]. Therefore, this stress-dependent stimulation of HO-1 expression is considered to be a component of the antioxidant network, with redox cycling between biliverdin and bilirubin as a mechanism for scavenging of ROS, such as lipophilic peroxyl radicals [7]. 

Upon degradation of the HO-1 substrate heme, CO is also generated. It is generally assumed that CO is a gaseous signaling molecule, which may play a role in the stress response and is involved in the cytoprotective events related to HO-1 activation in addition or alternatively to biliverdin/bilirubin redox cycling. One of the main intracellular targets of CO is the mitochondrial respiratory chain, which is in particular inhibited by the binding of CO to the heme moiety of cytochrome *c* oxidase [33,34]. Inhibition is accompanied by an elevated generation of ROS, especially superoxide formation via one-electron transfer to oxygen [19,35]. At low levels, deliberately generated ROS are also considered to be signaling molecules activating downstream proteins by targeted oxidation [36].

Early events of CO signaling aimed at the respiratory chain can be studied by means of extracellular flux technology and the use of CO-generating systems. These CO-releasing molecules (CORMs) are suitable tools to ensure a continuous supply of intracellular CO and can thus be used to mimic endogenous CO production. As determined with this methodology, the expected inhibition of the respiratory chain after exposure of cells to the CO donor CORM-401 was observed at time points later than 60 min (Figure 1A). 

A more detailed analysis of CO-mediated modulation of cellular respiration in the mito stress test revealed a specific pattern of CO-dependent effects comprising a decreased maximal respiration, lowered ATP production, together with an increased proton leakage (Figure 2). These characteristics were found to be very similar in different cell types (Figure S2) and are in accordance with the literature. CORM-401 was found to decrease mitochondrial ATP turnover and maximal respiration while increasing proton leakage in endothelial EA.hy926 cells and 3T3-L1 adipocytes as determined with extracellular flux technology [37,38]. Taken together, the present results underline the validity of our test system, which was further used in the study to investigate the role of HO-1 expression and substrate dependency on endogenous CO production.

In order to study the impact of endogenous CO formation on mitochondrial respiration, we modulated HO-1 protein levels via overexpression and gene silencing. Hemin was applied as a substrate for heme oxygenases and cells were subjected to the mito stress test. Very similar to the observations made with CORM-401 treatment, a decrease in maximal respiration and ATP production was found whereas proton leakage was increased (Figure 3), which is in accordance with the literature [39], thus indicating that HO-dependent production of endogenous CO modulates mitochondrial respiration. Under the conditions applied here, the modulation of mitochondrial respiration was dependent on the amount of the substrate hemin but not on the expression level of HO-1. Therefore, we suggest that availability of the substrate plays a role in the regulation of heme oxygenase-mediated CO signaling. 

Hemin was also recently described to exert neuroprotective effects via abrogation of sevoflurane-induced activation of the mitochondrial intrinsic pathway of apoptosis [40]. This was discussed to be mediated via induction of neuroglobin levels; however, CO signaling following hemin degradation by heme oxygenases was not excluded as the mode of action. Interestingly, another metabolite of heme degradation (bilirubin) was also found to modulate mitochondrial function [41]. The authors showed that after long exposure (24 h) of murine adipose tissue to bilirubin, OCR levels were increased. This was discussed to be mediated via binding of bilirubin to peroxisome proliferator-activated receptor-α (PPARα) and subsequently an increased mitochondrial biogenesis. Such a long-term effect probably complements the quicker and more direct regulation of mitochondrial function by CO signaling demonstrated here.

Typically, an inhibition of respiration is associated with less ATP production and a subsequent compensation via increased glycolytic flux, which we also observed as an elevated acidification rate at later time points (>60 min) following treatment with CORM-401 (Figure 1B). However, directly after treatment with CORM-401 (up to 30 min), glycolysis was decreased and stayed below the control level (Figure 1B), suggesting a direct regulatory effect of CO on intracellular glucose utilization, which was further confirmed by the glycolytic rate assay (Figure 4A). Here, compensatory glycolysis was also less pronounced upon CORM-401 exposure (Figure 4C). Glycolysis enzyme levels were excluded as the limiting factor. 

We therefore followed the metabolic fate of glucose after CORM-401 administration using [U-^13^C]-glucose for isotopic tracing (Figure 5). The labeling pattern basically confirmed the extracellular flux data, providing evidence for a transient decrease of glycolysis at early time points (<30 min) followed by an increased flux at later time points (>60 min). 

The transient decrease of glycolysis directly after CO exposure is accompanied by a short prominent burst of a flux through the PPP, redirecting metabolites back towards glycolysis via G3P. Such an induction of the PPP by CO has been reported [26,42]; however, we highlight here the dynamic nature of this effect and its interplay with energy-providing glucose metabolism, which has not been described so far. Redirection of PPP metabolites indicates that the major goal of this shift is related to the provision of the reduction equivalent NADPH as an essential element of antioxidant defense. It is interesting to note that this also includes the redox cycling system provided by biliverdin and bilirubin, suggesting that endogenous CO generated by heme oxygenases can mediate a rapid feed-forward stimulation for this pathway. Concomitantly, ROS generation was shown to be elevated after CORM-401 exposure. These observations may shed light on contradictory reports about pro- and antioxidative properties of CO [13,43] and suggest an additional role for heme oxygenases as part of a complex antioxidant network.

## 5. Conclusions

The present data add further evidence that endogenously generated CO plays a role in cellular stress response. Upon regulation of glucose metabolism by CO, reduction equivalents (NADPH) for antioxidant defense are provided on short term, whereas inhibition of mitochondrial respiration and shift to glycolysis is a later effect. CO signaling mediated by HO activity is in part substrate-regulated and likely complements antioxidant defense, provided by the HO-dependent biliverdin/bilirubin redox cycling system.

## Supplementary Materials

The following are available online at https://www.mdpi.com/2076-3921/9/8/652/s1, Figure S1: Schematic depiction of OCR curves from Figure 2A and the resulting calculated parameters, Figure S2: CO-derived effect pattern of mitochondrial respiratory parameters is similar in different cell types, Figure S3: Cyanide application leads to an inhibition of maximal respiration and ATP production, but no increase of proton leakage, Figure S4: Genetical modification of HO-1 protein levels in MEFs, Table S1: Statistical analysis belonging to Figure 2B–D, Table S2: Statistical analysis belonging to Figure 3, Table S3: Statistical analysis belonging to Figure 4B,C, Table S4: Statistical analysis belonging to Figure 5.
