# Supplementary material for: Endogenous Carbon Monoxide Signaling Modulates Mitochondrial Function and Intracellular Glucose Utilization: Impact of the Heme Oxygenase Substrate Hemin
Source: Antioxidants (Basel). 2020 Jul 23;9(8):652. doi: 10.3390/antiox9080652 (PMC7465082; doi:10.3390/antiox9080652)
Supplement: Supplementary file 1 [file antioxidants-09-00652-s001.pdf]

Supplementary material of:

Endogenous carbon monoxide signaling modulates mitochondrial function and intracellular glucose utilization: impact of the heme oxygenase substrate hemin.

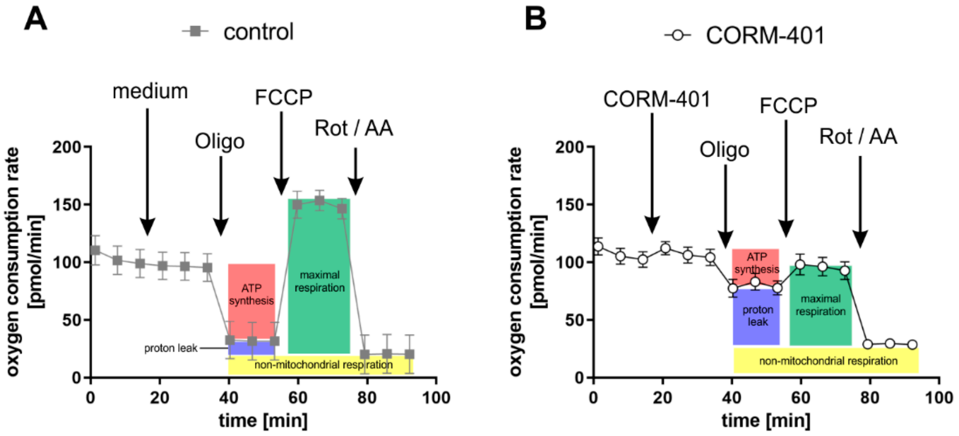

Figure S1: Schematic depiction of OCR curves from Fig 2A and the resulting calculated parameters. Red: ATP synthesis, blue: proton leak, green: maximal respiration, yellow: non-mitochondrial respiration.

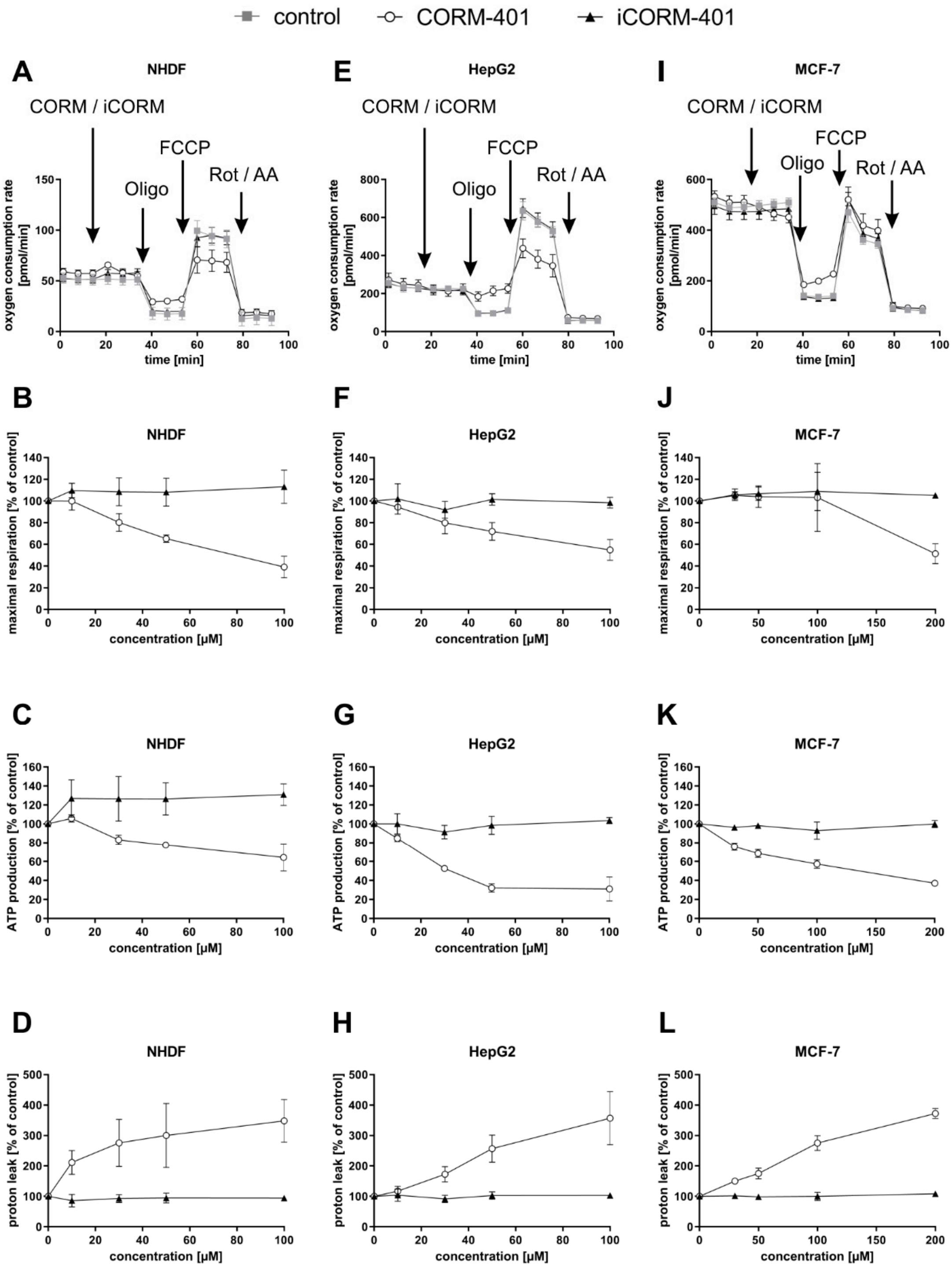

Figure S2: CO-derived effect pattern of mitochondrial respiratory parameters is similar in different cell types. CORM-401 and iCORM-401, were injected into reaction wells and the mito stress test (sequential injection of oligomycin, FCCP and rotenone + antimycin A) was performed. Representative OCR curves at the level of 50 µM CORM-401/iCORM-401 are given for NHDF (A), HepG2 (E) and MCF-7 (I) cells. Quantifications of the mitochondrial respiratory parameters (maximal respiration, ATP production and proton leak) for the different cell types are given: NHDF (B-D), HepG2 (F-H), MCF-7 (J-L). Data represent mean ± SD of three independent experiments (n = 3). OCR = oxygen consumption rate, NHDF = normal human dermal fibroblasts, HepG2 = human hepatocellular carcinoma cells, MCF-7 = human mammary gland breast cancer cells.

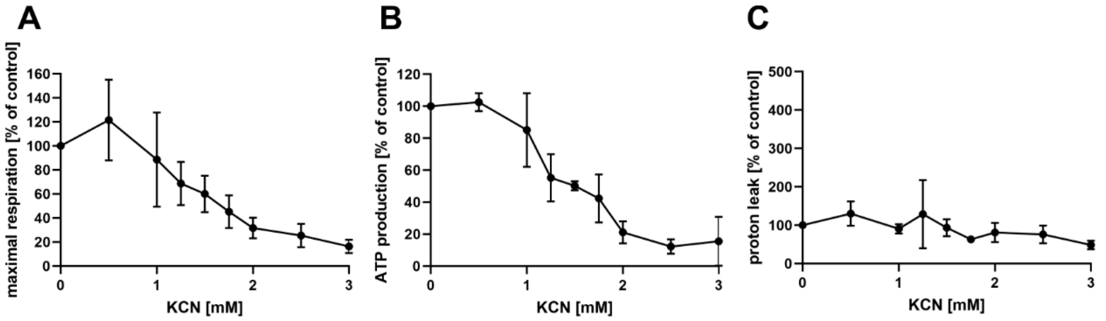

Figure S3: Cyanide application leads to an inhibition of maximal respiration and ATP production, but no increase of proton leakage. KCN was injected into reaction wells and the mito stress test (sequential injection of oligomycin, FCCP and rotenone + antimycin A) was performed. Quantifications of the mitochondrial respiratory parameters (maximal respiration (A), ATP production (B) and proton leak(C)) are given. Data represent mean ± SD of three independent experiments (n = 3).

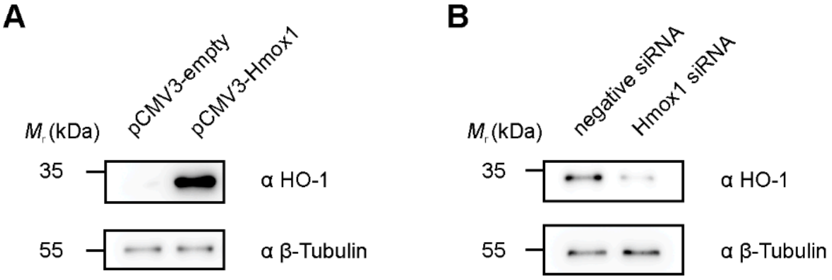

Figure S4: Genetical modification of HO-1 protein levels in MEFs. Representative Western blot analyses of HO-1 protein levels of MEFs (n = 3). Overexpression of HO-1 was achieved by transfecting cells with pCMV3-Hmox1 (A). As control pCMV3-empty (empty vector) was used. Knock down of HO-1 was achieved by transfecting cells with siRNA directed against Hmox1 (B). As control negative siRNA was used.

Table S1: Statistical analysis belonging to figure 2B-D. For statistical analysis one-way ANOVA was used with Sidak's multiple comparisons test. **p*<0.05, ***p*<0.01, ****p*<0.001, n.s. = not significant, ct = control.

parameter	ct vs CORM-401				ct vs iCORM-401				CORM-401 vs iCORM-401				
	10	30	50	100	10	30	50	100	10	30	50	100	μM
maximal respiration	n.s.	n.s.	**	***	n.s.	n.s.	n.s.	n.s.	n.s.	n.s.	*	***	
ATP production	n.s.	*	***	***	n.s.	n.s.	n.s.	n.s.	n.s.	*	***	***	
proton leak	n.s.	***	***	***	n.s.	n.s.	n.s.	n.s.	n.s.	***	***	***	

Table S2: Statistical analysis belonging to figure 3. For statistical analysis one-way ANOVA was used with Sidak's multiple comparisons test. Untreated (0 μM hemin) empty vector group was used as reference. **p*<0.05, ***p*<0.01, ****p*<0.001, n.s. = not significant, ct = control, ref = reference.

group	hemin [μM]	maximal respiration	ATP production	proton leak	group	hemin [μM]	maximal respiration	ATP production	proton leak
empty vector	0	ref	ref	ref	negative siRNA	0	ref	ref	ref
	2	n.s.	n.s.	n.s.		2	**	n.s.	*
	5	n.s.	***	*		5	n.s.	**	**
	10	n.s.	***	n.s.		10	**	***	**
	20	n.s.	***	*		20	**	***	***
HO-1	0	n.s.	n.s.	n.s.	*Hmox1* siRNA	0	n.s.	n.s.	n.s.
	2	n.s.	n.s.	n.s.		2	***	n.s.	**
	5	n.s.	n.s.	n.s.		5	n.s.	**	*
	10	n.s.	***	n.s.		10	**	***	**
	20	n.s.	***	*		20	**	***	***

Table S3: Statistical analysis belonging to figure 4B+C. For statistical analysis one-way ANOVA was used with Sidak's multiple comparisons test. **p*<0.05, ***p*<0.01, ****p*<0.001, n.s. = not significant, ct = control.

parameter	ct vs CORM-401				ct vs iCORM-401				CORM-401 vs iCORM-401				
	10	30	50	100	10	30	50	100	10	30	50	100	μM
induced glycolysis	n.s.	*	**	**	n.s.	n.s.	n.s.	n.s.	n.s.	**	***	***	
compensatory glycolysis	n.s.	**	***	***	n.s.	n.s.	n.s.	n.s.	n.s.	**	***	***	

Table S4: Statistical analysis belonging to figure 5. For statistical analysis one-way ANOVA was used with Sidak's multiple comparisons test. *p<0.05, **p<0.01, ***p<0.001, n.s. = not significant, ct = control.

parameter	ct vs CORM-401			ct vs iCORM-401			CORM-401 vs iCORM-401			
	30	90	240	30	90	240	30	90	240	min
G6P M+6	***	***	***	*	n.s.	n.s.	*	**	***	
F1,6BP M+6	***	***	***	n.s.	n.s.	n.s.	***	***	***	
G3P M+3	***	***	***	*	n.s.	n.s.	***	***	***	
1,3BPG M+3	n.s.	**	***	n.s.	**	**	n.s.	***	***	
PEP M+3	**	*	n.s.	n.s.	**	**	n.s.	n.s.	*	
6PG M+6	***	***	***	***	n.s.	n.s.	***	***	***	
Ru5P M+5	***	***	**	**	n.s.	n.s.	n.s.	**	**	
S7P M+7	***	***	***	***	n.s.	n.s.	***	***	***	
E4P M+4	***	***	***	*	n.s.	n.s.	***	***	***	
PRPP M+5	n.s.	n.s.	***	**	n.s.	n.s.	n.s.	n.s.	***	
